# Including business strategy in model-driven methods: an experiment

**DOI:** 10.1007/s00766-023-00400-3

**Published:** 2023-03-10

**Authors:** Rene Noel, Jose Ignacio Panach, Oscar Pastor

**Affiliations:** 1 Valencian Research Institute for Artificial Intelligence, Universitat Politècnica de València, Camino de Vera s/n, 46022 Valencia, Spain; 2 Escuela de Ingeniería Informática, Universidad de Valparaíso, Valparaíso, Chile; 3 Escola Tècnica Superior d’Enginyeria, Universitat de València, Valencia, Spain

**Keywords:** Organisational modelling, Goal modelling, Model-driven architecture, Business strategy modelling, Organisational structure modelling

## Abstract

Software-centric organisations design a loosely coupled organisation structure around strategic objectives, replicating this design to their business processes and information systems. Nowadays, dealing with business strategy in a model-driven development context is a challenge since key concepts such as the organisation’s structure and strategic ends and means have been mostly addressed at the enterprise architecture level for the strategic alignment of the whole organisation, and have not been included into MDD methods as a requirements source. To overcome this issue, researchers have designed the LiteStrat, a business strategy modelling method compliant with MDD for developing information systems. This article presents an empirical comparison of LiteStrat and with i*, one of the most used models for strategic alignment in an MDD context. The article contributes with a literature review on the experimental comparison of modelling languages, the design of a study for measuring and comparing the semantic quality of modelling languages, and empirical evidence of the LiteStrat and i* differences. The evaluation consists of a 2 × 2 factorial experiment recruiting 28 undergraduate subjects. Significant differences favouring LiteStrat were found for models’ accuracy and completeness, while no differences in modeller’s efficiency and satisfaction were detected. These results yield evidence of the suitability of LiteStrat for business strategy modelling in a model-driven context.

## Introduction

The Object Management Group (OMG) has proposed an architecture for model-driven development (MDD) named model-driven architecture (MDA) [1]. MDA divides the system into several abstraction layers; the most abstract is the computation-independent models (CIMs). CIM aims to represent business-related concerns, such as business goals, processes, and commercial models, among many others [2–4]. Through model-to-model transformations, CIMs are transformed into platform-independent models (PIM) and then to platform-specific models (PSM), providing traceability and consistency between business concerns and the information system design. Moreover, model-to-model transformations at the CIM level have also been exploited to provide strategic alignment of business goals and processes [5–7], in order to improve information system requirements. Hence, representing critical business information for the design of strategically aligned information systems is crucial for model-driven initiatives.

In the last years, new business concerns have gained relevance in the design and development of information systems. Recent research on software-centric organisations (SCO) has found that independent, cross-functional organisation units (agile teams, agile cells, squads, among other names), which are aligned to business strategy, are more efficient in delivering software [8]. This finding is predicted by Conway’s law, which states that organisations replicate their structure to everything they design [9]. The approach of designing an autonomous, loosely coupled, and business-oriented organisational structure has been taken into account by many frameworks for scaling agility across the enterprise [10–13]. These frameworks recommend continuously evolving the organisational structure according to the desired information systems design [8]. Moreover, broadly adopted approaches guide developers in designing strategically aligned software components according to the specific context of the different organisation units [14, 15].

In a model-driven context, supporting SCO’s practices for strategic alignment is challenging since it requires modelling the organisation’s ends and means and organisational structure concepts to trace them to the information system requirements.

In conceptual modelling, strategic alignment has been addressed by two main approaches: enterprise architecture (EA) and by integrating goal modelling into MDA-based initiatives. On the one hand, business strategy has been conceptualised in EA frameworks; in particular, ArchiMate [16] and the Business Motivation Model (BMM) [17] have concepts to represent the high-level goals and courses of action; BMM has concepts for strategy and goals and more specific tactics and objectives. However, EA frameworks are focused on the alignment of the whole organisation’s IT strategy [18] and do not cover the detailed specification of the information system (needed to support model-driven development) or consider organisational structure concepts.

On the other hand, goal-oriented modelling frameworks have been successfully used for aligning model-driven methods with the system stakeholders’ goals [6, 19–21]. Notably, i* [22], has been applied for organisational modelling [23], and its social constructs could be used for representing organisational structure [24]. However, i* lacks some business strategy concepts [25], and while there are proposals for systematically designing i* models [26], it does not provide support to determine which modelling constructs to use [27]. These facts hinder modelling business strategy and integrating i* models with other models.

In the previous work, researchers have designed LiteStrat [28], in order to address the above challenges. LiteStrat is a business strategy modelling method designed through the careful selection of concepts and relationships from i* and from EA frameworks, in particular, ArchiMate’s motivation and strategy elements [16], and the Business Motivation Model’s concepts for defining high-level and more specific organisational ends and means [17]. LiteStrat adds the *organisation unit* concept to support jointly modelling business strategy and organisational structure and a modelling procedure to foster the integration with other models in an MDA context. LiteStrat was designed to introduce business strategy and organisational structure information at the CIM level of MDA-based methods as an input for model-to-model transformations to the PIM and PSM levels to provide traceability and as much automation as possible for developing strategically aligned systems. Besides its applicability to requirements engineering, LiteStrat has a specific target on the model-driven development of software systems. On the one hand, it introduces the organisation structure to support the approach of SCO for designing software components; on the other hand, the proposed modelling procedure aims to ensure the correct delegation of goals to organisational roles, which is needed by most model-driven frameworks which aim for the automatic software code generation [3, 6, 7, 20]. The central claim of LiteStrat is that it provides a more accurate and complete way to capture business strategy knowledge than existing goal modelling frameworks, since it was designed with that purpose. However, adding a tighter modelling procedure could hinder modellers’ efficiency and satisfaction.

Since i* is one of the most studied goal-oriented requirements engineering frameworks [29] and is also part of a family of goal modelling languages that have been applied for strategic alignment of business processes in model-driven methods [30], such as GRL [7] and TROPOS [20], attempts have been made to extend it to include organisational information in requirements analysis [52]; the comparison between LiteStrat and i* is relevant for researchers of strategic alignment of model-driven methods and requirements engineering. This article presents an experimental comparison of LiteStrat and i* on modelling business strategy. The article’s contribution is threefold. Firstly, it provides a literature review on experimental comparisons of modelling languages. Secondly, the article presents in detail an experimental design and measurement approach for the semantic comparison of two modelling languages. Finally, the article provides empirical evidence on whether LiteStrat, a specifically designed method, can improve a more generalist one such as i* for representing business strategy and organisation structure concepts.

We conducted an experiment with 28 subjects from the Universidad de Valparaíso, in Chile. Using a 2 × 2 experimental design, with the modelling method as a factor (i* and LiteStrat), and two different experimental problems to block its influence, the subjects performed a modelling activity to represent a business strategy case in a LiteStrat or i* model. The response variables were the accuracy and completeness of the models and the efficiency and satisfaction of the users. The experiment results show that LiteStrat’s claims about capturing business strategy more accurately and completely were experimentally verified with statistical significance. No differences in user efficiency and user satisfaction were found.

The rest of the article continues with Sect. 2, which covers a review of works on experimental comparisons of modelling methods and the study’s experimental design. Section 3 describes the results of the experiment, while Sect. 4 presents the discussion and the final conclusions.

## Experimental comparison of LiteStrat and i*

### Related works on experimental comparisons of modelling methods

In order to select the measurement procedure and metrics for the study, we reviewed the existing experimental comparisons of modelling methods, languages, tools, or notations with respect to the quality of the resulting models. Inspired by Petersen’s guidelines [31], we conducted a targeted literature review. We searched for experimental comparisons of modelling methods in Web of Science, using the following search string: (TS = ((experiment* OR empiric*) AND (comparis* or evaluation or assess* or ‘versus’ or ‘vs’) AND model* AND conceptual and (language* or method* or tool*) AND (< *modelling method*>)), where the modelling methods considered were organisational, strategy, goal, business process, and class models. The resulting 224 papers were filtered applying the following inclusion (IC) and exclusion criteria (EC): (IC1) studies must refer to experiments and not to other empirical approaches; (IC2) the object of the study must be modelling languages, methods, notations, or modelling tools, and not to other objects such as algorithms or transformation techniques; (EC1) the design of the activity must focus on creating the models; (EC2) at least one of the assessed variables must regard to how well the method represents the problem domain. Thirty-two articles were selected after the title and abstract review, and eight were finally selected after reviewing the full article.

We performed a backward snowballing [32] looking for relevant referenced studies, adding five more articles. From the resulting thirteen studies, we examined the compared methods, languages or tools, the number of participants and whether they are professionals or students. Our primary focus was to analyse the metrics and measurement procedures for assessing quality, and the conclusions of the experimental comparisons. Table 1 presents the main findings. Next, we summarise and classify our findings according to their approach to quality measurement.

**Table 1:** Summary of related works

Study	Method, language, or tool	Participants	Variables and metrics	Results
[34]	i*, value@GRL	40 M.Sc. students, 28 software engineers, 12 analysts	Correctness, Completeness, Efficiency, Perceived ease of Use, Productivity	Value@GRL produces models of higher quality, and users are more productive. Also, value@GRL is perceived to be easier to use
[35]	i*, value@GRL	100 M.Sc. students, 64 UG students, 20 professionals	Model Quality, Efficiency, Productivity. Perceived ease of Use, Productivity	Value@GRL is more efficient, productive, and is perceived to be more useful by users. Also, value@GRL produces models with better quality
[36]	Context-aware Feature Model, Tropos	10 Ph.D. and M.Sc. students	Effectiveness, Efficiency	Context-aware Feature Model is more effective than Tropos Goal Model considering precision, but no differences in recall were found
[37]	Think-Pair-Square, Face to Face	36 UG and M.Sc. students	Correctness, Completeness, Time	Face-to-Face interaction is less time-consuming, and no significant differences in quality were observed
[38]	FOOM, POOM	156 undergraduate (UG) students	Correctness, Comprehension, Efficiency, Preference	FOOM specifications are more correct, comprehensible, and preferred by the users
[39]	OPM, OMT	86 UG students	Correctness, Comprehension	OPM produces specifications with higher quality and is more comprehensible
[40]	Entity-Relationship, Object Oriented	44 UG students	Correctness, Time, Preference	EER yields to better modelling quality regarding unary and ternary relationships, is less time-consuming, and is preferred by users
[41]	Business Process Models, Use Cases	75 UG students	Accuracy, Dependency understandability, Integration understandability	BPMN specifications produce better understanding of the execution order dependencies. No differences in Accuracy and Integration Understandability were found
[42]	ENSURE, Traditional RE	18 post doc and professional subjects	Effectiveness, Efficiency, Practicability	ENSURE is more effective, and efficient and is perceived to be more practical than a traditional RE approach
[43]	BPRIM diagram, multi-view tool	41 bachelor, M.Sc., and Ph.D. students	Correctness, Usability, Efficiency	Multi-view modelling is more usable and efficient and produces more correct models
[44]	Tangible modelling, Computer-aided modelling	8 UG students	Correctness, Learnability, Efficiency, Satisfaction	The results suggest that Tangible Modelling is faster, easier to learn, more efficient, and produces more correct models and more satisfaction to the users
[46]	i*, KAOS	19 graduate students	Model Quality, Language Quality	The KAOS language quality is higher than i*. i* models have higher quality than KAOS models
[47]	ERD, Class Diagrams, Natural Language	103 UG students	Correctness, Time, Perceived Ease of Use	ERD specifications are easier to understand, more helpful, and less time-consuming for use case modelling

#### Information Retrieval Approach

The information retrieval (IR) approach is based on the work of Frakes and Baeza-Yates [33]. It is focused on measuring models’ completeness (containment of all the information) and correctness (conformance with the modelled domain) through the precision and recall metrics. Precision is the number of correctly modelled elements compared to an ideal model (the oracle) out of the total number of modelled elements. Recall refers to the number of correctly modelled elements out of the total elements in the oracle. Different precision and recall metrics are usually defined for every construct of the modelling language.

Abrahao et al. applied IR for assessing two goal-modelling languages (i* and their proposal, value@GRL) for incremental software development [34]. The researchers summarised the quality in a single metric (F-measure) as the harmonic mean of the precision and recall metrics for the quality evaluation. Significant results favoured value@GRL over i*. The study was replicated and reported as a family of experiments in [35], preserving the experimental design and involving 184 subjects. The experimental replications confirmed the initial results.

The study by Jesus Souza et al. [36] compares two modelling techniques for Dynamic Software Product Lines: the Context-aware Feature Model and the Tropos Goal Model. Significant precision differences were found. Scanniello and Erra [37] also applied the IR approach to study the quality of models produced by two requirements engineering modelling approaches: their proposal, Think-Pair-Square, and the Face-to-Face approach. The research presents two replications of the same study involving 36 bachelor and master students, and no significant differences in model quality were found. The authors discuss that creating an oracle model could introduce bias because the oracle model is just one of many possibilities to represent a problem description, which was mitigated by pair-reviewing the oracles. This threat to validity is not discussed in the other works mentioned above.

#### Semantic quality inspection approach

Semantic quality inspection is the name we give to the expert review of the models produced by the subjects with the source specification document. We found two types of metrics: a score based on a grading scheme and a simple count of errors or hits. Kabeli and Shoval [38] compared two analysis specification methodologies to assess the correctness of the resulting model: their proposal, functional and object-oriented methodology (FOOM) and object-process methodology (POOM), with a total of 156 participants. The grading scales defined different error points depending on the severity of the error. Significant differences in quality were found favouring FOOM. The same approach is applied by Peleg and Dori [39] for comparing their modelling method proposals, object-process methodology (OPM) and object modelling technique (OMT), in order to assess their correctness. The experiment involved 86 participants. The grading scheme has 38 items, each of which can have a score from 0 to 1, with minor, medium, and major errors with 0.25, 0.5, and 0.75 points, respectively. Of the 38 items, 8 presented significant differences favouring OPM and 2 favouring OMT. The authors refer their selection of the grading scheme to a previous work by Shoval and Shiran [40], who compare two data modelling techniques: extended entity relationship (EER) and object-oriented (OO). The grading scheme had nine items, finding significant differences for two items, favouring EER.

Trkman et al. [41] compared the effects in user stories’ identification of two business process specification formats: business process models and use cases. The researchers counted the correctly identified stories, having as subjects 75 undergraduate students; no significant differences were found. Saputri and Lee [42] compared the effectiveness of two sustainability requirements engineering (RE) methods: the traditional RE approach and their proposal, ENSURE, in a study that involved 18 experienced subjects. Effectiveness was measured by counting the number of correctly identified stakeholders, requirements, features, and the percentage of solved conflicts, with significant results favouring their proposal.

Thabet et al. [43] compared two tools that support the business process-risk management-integrated method (BPRIM): a multi-view tool and a diagram-oriented one. The researchers assessed the correctness of the models produced by 41 subjects by counting the errors against the source specification. The authors counted the number of modelling errors (wrong use of constructs) and semantic errors (when the subjects miss elements of the underlying business process model). The results for the two measurements showed significant differences favouring the multi-view tool. Ionita et al. [44] studied the effects of tangible modelling on eliciting domain knowledge. Tangible and computer-aided designed models were assessed by counting errors, considering three types of errors: (1) placing an element where none was expected; (2) a missing element where one was expected; and (3) using a wrong concept to represent an element. The tangible group finished 52% faster and had 50% of the errors than the control group.

The semantic quality inspection approach introduces subjectivity, which is usually mitigated by the collaborative definition of the grading scales and error types.

#### Other approaches for model quality measurement

Another approach for comparing models’ quality is applying the semiotic approach using the SEQUAL framework by Krogstie [45]. In [46], Matulevičius and Heymans applied it to compare the quality of two goal-modelling languages using a 5-points Likert scale for 15 questions derived from the quality attributes proposed in SEQUAL. Another approach is to directly count the number of mismatches of the models produced by the subjects and an ideal model (gold standard), which is followed by Ibriwesh et al. [47] to compare three data documentation perspectives as inputs for use-case modelling.

#### Efficiency and Satisfaction Measurement

As summarised in Table 1, besides the quality of the resulting models, most experimental comparisons also measure the users’ efficiency and satisfaction, usually to assess whether there are unintended effects of new modelling languages or tools. Efficiency is mostly measured by the time needed to complete a modelling task [36–38, 40, 42–44, 47]. With regard to user satisfaction, in [44], the authors considered the usability questionnaire proposed by Lewis [48], while Saputri et al. defined an ad hoc instrument of 27 Likert scale questions [42] for assessing practicality and usability. Abrahao et al. [34, 35] measured the modellers’ satisfaction by the perceived ease of use and perceived usefulness, based on the technology acceptance model by Davis [49]. Matulevicius and Heymans [46] designed a questionnaire based on the pragmatic quality attributes of the SEQUAL framework.

In summary, the comparison of the models’ quality is mainly assessed in terms of correctness and completeness, and the inspection of models against the original specification over a grading scheme is one of the most used measurement approaches. Other complementary variables are user efficiency and user satisfaction, measured in terms of users’ perception of ease of use and usefulness of the modelling methods.

### Background: organisational modelling with i* and LiteStrat

The study focuses on comparing two modelling methods for representing organisational level information regarding business strategy and organisational structure: i* and, LiteStrat. i* [22] is a general-purpose goal modelling framework that has been broadly used for requirements and software engineering, but also for organisational modelling [23]. Although i* is not an organisational modelling method, it is arguably one of the best alternatives for including organisational information into the CIM level of an MDA-based method. Indeed, many initiatives have applied i* at the business strategy level [50–52], and even some extensions have been proposed for adding organisational modelling constructs, as reviewed by Goncalves et al. [53]. Importantly, i* has been considered at the CIM level of MDA-based initiatives for strategic alignment [6, 7, 54], but mostly for representing stakeholders’ goals regarding the system, and not for representing business strategy or organisational structure. However, i* constructs can be used to model these latter concerns, as explored in a proposal for systematically modelling business strategy in an MDA context [24].

On the other hand, LiteStrat [28] is partially based on i* and was designed explicitly to model business strategy and organisational structure to include this information into MDA-based software development processes. Below, we present the two methods and a comparison of their constructs.

#### The i* framework

i* [22] is an agent and goal-oriented modelling language proposed by Eric Yu in 1995. For our experiment, we consider the reviewed version by Dalpiaz et al., iStar 2.0 [55]. For simplicity, we will refer to this latest version as i* in the rest of the paper.

The main construct of i* is social dependency, which is the relationship between an actor who needs to achieve a goal and another actor who can help her or him achieve the goal. These dependencies are represented in the *Strategic Dependency* diagram, which allows different types of actors and dependencies to be connected. *Agents* are actors that represent real-world subjects, such as organisations, departments, or a specific person, and *roles* are a characterisation of behaviours of social actors. The different types of dependencies are *goal*, where the depending actor completely delegates the goal achievement as well as the know-how to achieve it, *task*, where the actor wants a task to be accomplished without delegating the know-how, *resource*, where the actor wants to attain a resource, and *quality*, which defines a quality that an actor needs to be fulfilled. Besides the dependencies, actors can also be related by the *participates-in* relationship, which, for instance, allows specifying if a role participates in a department (represented by an agent).

It is also possible to specify the goals, qualities, resources, and tasks that the actors need to perform to fulfil their social dependencies. These inner elements are specified in the *Strategic Rationale* diagram. There are different relationships among the actors’ inner strategic elements. *Refinements* allows decomposing tasks or relating a goal to the tasks needed to achieve it; the *needed-by* relationship supports specifying the resources needed for a task; the *qualify* relationship associates a quality to an element; and the *contribution* relationship defines how an element contributes to a goal.

It is worth noting that i* does not specify guidelines for the modelling activity. Researchers have proposed using i*’s goals and tasks to specify strategic ends, means, i*’s agents, roles, and the ‘participates-in’ relationship to represent the organisational structure [24]. We will consider this initiative for training the experimental subjects not as a prescriptive modelling procedure but as examples of the feasibility of using i* for modelling business strategy.

#### LiteStrat

LiteStrat [28] is an organisational modelling method that proposes a systematic approach for modelling business strategy. LiteStrat is partially based on i*, but integrates constructs and a modelling approach from other modelling frameworks [16, 17]. LiteStrat applies i* modelling approach for representing strategic elements and organisational structure that provides: (1) a modelling language to represent business strategy and (2) a modelling procedure to apply the language systematically. The goal of LiteStrat is to better support the collection and connection of business strategy information in an MDA context by reducing ambiguity through its more precise business strategy constructs and modelling guidance.

Similarly to i*, LiteStrat’s language considers social actors and intentional elements. For social actors, the *Actor* construct represents entities outside the organisation whose behaviour cannot be described more deeply than by their actions from the perspective of the organisation under analysis. *Organisation Units* represent the organisation or its units, such as departments or teams. *Roles* characterise behaviours in a given context. Concerning the relationships among actors, organisation units can be part of other organisation units, and roles can be part of organisation units. In contrast to i*, LiteStrat proposes a special type of link between actors and organisation units in order to represent the behaviours of an actor or unit that are not intentionally directed towards another actor or unit, but that nonetheless affect their internal strategic definitions: the *influence* relationship. This link is suitable for relating the organisation or its units with actors whose internal strategic elements cannot be accessed, such as competitors, customers, or other organisation units outside the analysis context. The intention constructs are the *goal*, which is a high-level desired state of affairs, and the *objective*, a more specific, measurable desired state. Regarding strategic actions, the *strategy* construct represents high-level actions towards achieving a goal, while *tactics* represent more specific and process-focused actions to implement the strategy.

LiteStrat specifies a modelling procedure based on an outside-in perspective for business strategy, providing guidelines to refine high-level goals and strategies to more specific tactics and objectives:

- The external actors and their influence on the organisation are identified. One or more overarching goals in the context of these influences are modelled.
- Then, a set of strategies for achieving the goal are modelled and then refined into one or more tactics, assigning them to the organisation units with the capabilities for implementing them.
- The tactics are then refined into objectives and assigned to specific roles accountable for their implementation.
- The expected outcomes of each organisation unit are modelled as influences on external actors (such as customers or providers) and other organisation units. Also, further external actors and their influence are added.

As a summary, Table 2 presents a comparison between i* and LiteStrat constructs.

**Table 2:** Differences between i* and LiteStrat constructs

i* construct	LiteStrat construct	Comment
*Actor types*		
Actor	Actor	In i*, Actors represent any type of intentional actor. In LiteStrat, Actors represent intentional actors that are outside the organisation and whose intention cannot be known
Agent	Organisation Unit	In i*, Agents represent an actor with concrete physical manifestation, such as an individual, organisation or department. In LiteStrat, Organisation Units represent the same elements except for individuals
Role	Role	Both are abstract characterisations of the behaviour of a social actor within some context
*Actor association links*		
Participates-in	Participates-in	While i* does not restrict the types of actors that can be linked, LiteStrat defines that only organisation units and roles can participate in organisation units
Is-A	–	While i* does not restrict the types of actors that can be linked, LiteStrat defines that only organisation units and roles can participate in organisation units
*Intentional elements*		
Goal	Goal	While both represent a desired state of affairs of any type of actor, in LiteStrat, it is reserved just for Organisation Units
	Objective	Is a LiteStrat’s specification of measurable goals that is reserved for Roles
Task	Strategy	While i* defines tasks as actions that an actor wants to be executed usually with the purpose of achieving some goal, LiteStrat separates these actions into high-level actions (Strategies) and specific actions (Tactics). Strategies represent an explicit high-level action towards the achievement of a goal
	Tactic	Represents concrete actions towards the implementation of a strategy
Quality	–	LiteStrat does not support the quality construct, since it is expected that objectives could serve to represent measurable desired levels of quality regarding the business strategy
Resource	–	Resource modelling is out of the scope of LiteStrat
*Social dependencies*		
Goal dependency	Objective assignment	In i*, any type of actor can socially depend on any other type of actor to achieve its goals. In LiteStrat, only Organisation Units can depend on roles to achieve objectives through objective assignment
Quality dependency	–	Not supported in LiteStrat
Task dependency	Tactic Assignment	In i*, any type of actor can socially depend on any other type of actor to achieve its goals in performing an action. In LiteStrat, only Organisation Units can depend on other Organisation Units to implement tactics
Resource dependency	–	Not supported in LiteStrat
–	Influence	In LiteStrat, Actors or Organisation Units can behave in a way that affects other Organisation Units or Actors, but not necessarily with an intention to affect them. LiteStrat proposes the Influence construct to represent this relationship
*Intentional element links*		
Refinement	Refinement	In i*, it is a hierarchical link between goals or tasks. In LiteStrat, it is also a hierarchical link, but the hierarchy is prescribed by the modelling procedure going from goals to strategies to tactics and then to objectives
Needed by	–	Not supported in LiteStrat
Contribution	–	Not supported in LiteStrat
Qualification	–	Not supported in LiteStrat

**Fig. 1:**
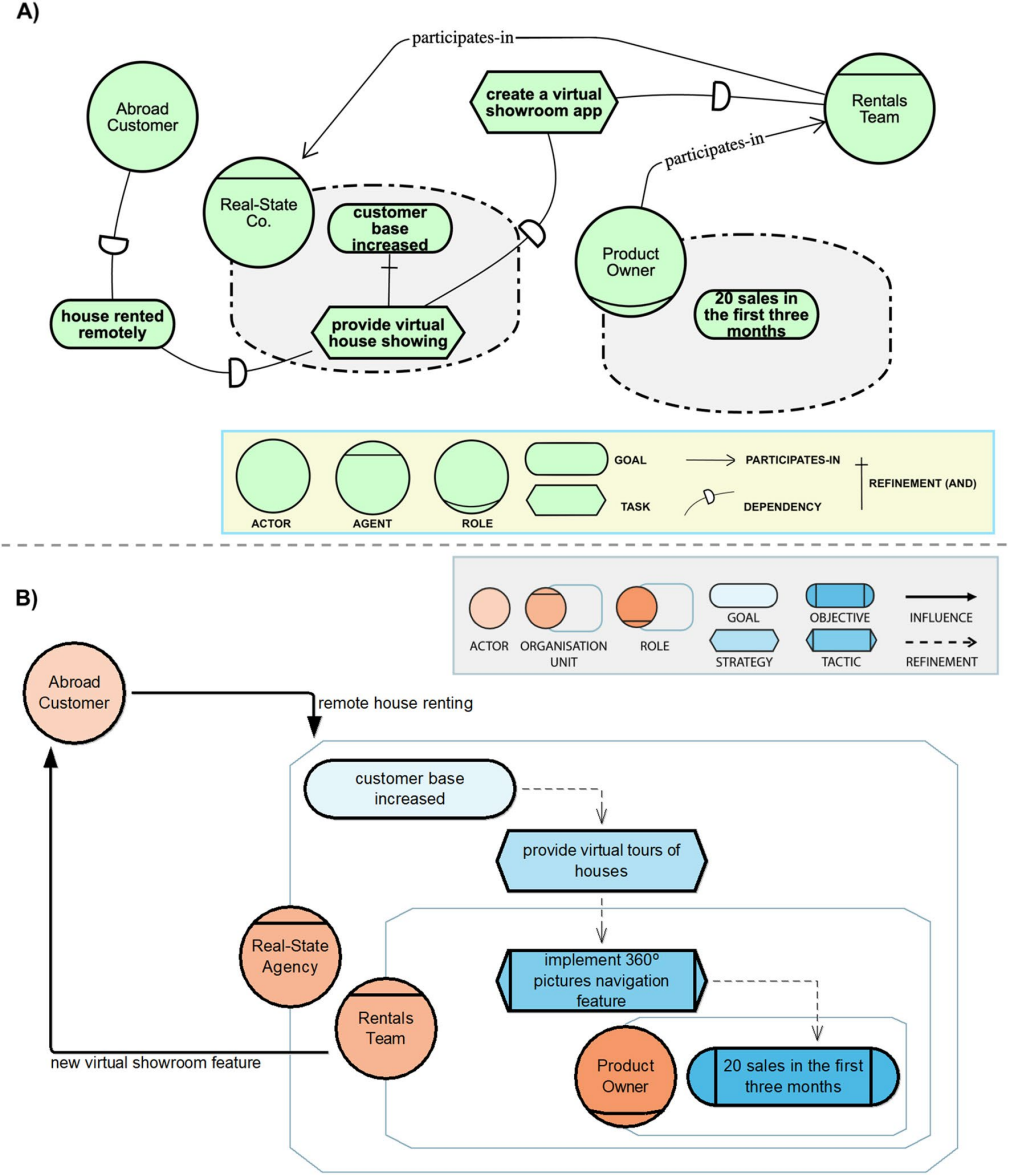
Examples of i* (**a**) and LiteStrat (**b**) for business strategy modelling

In Fig. 1, we provide examples on using i* (A) and LiteStrat (B) for business strategy modelling. Both diagrams depict the following strategic scenario: Real-Estate Co. is a house renting company that has detected the need of abroad customers to remotely rent a house. Under this scenario, the company sets the strategic goal of increasing its customer base and defines as its main strategy to provide virtual tour for the houses. Besides other specific actions needed to implement this strategy, the company defines that the Rentals Team must implement the virtual showroom by allowing users to navigate 360º pictures in the company’s app. The success of the strategy will be measured in terms of a specific objective: to rent 20 houses in the first three months since the feature is delivered. The responsible of tracking and reporting this objective is Rentals Team’s Product Owner.

In Fig. 1, some of the differences between the two modelling approaches are exemplified. For instance, in i* the same concept for expressing a desired situation (*goal*) can be used for both high-level intentions such as the company’s goal (“customer base increased”) and for the intention of an organisational role (“10 sales in the first three months”). On the contrary, LiteStrat provides different constructs (*goal* and *objective*) for these two different levels, which allows a clearer differentiation between them. Similarly, i*’s *task* construct is used to represent both high-level (“provide a virtual showroom") and specific actions (“implement the 360º pictures navigation feature”), which have different concepts in LiteStrat (*strategy* and *tactic*). Please note that the example is limited to show a specific type of difference and many other differences can arise from using all the constructs in Table 2.

### Experimental definition and planning

The experiment aims to compare i* versus LiteStrat for organisational modelling, specifically regarding business strategy. The purpose is to study the produced models’ quality as well as the quality of the modelling method. The research is designed from the perspective of modellers and researchers interested in representing business strategy for its inclusion in MDA-based development processes. The quality perspective of model users, e.g., stakeholders at the strategic level that should read the models, is considered a step after the validation presented in this article, and it is beyond the scope of this study.

The context of the experimental comparison is an MDA-based development process that starts by modelling the business strategy scenario that triggers changes in business processes and in the information systems that support those processes. In this context, the primary model quality perspective concerns whether i* or LiteStrat better represent business strategy information so it can be accessed from other models at the CIM, PIM, and PSM levels in an MDA context. A secondary model quality perspective in an MDA-based development process regards whether i* or LiteStrat models serve better to convey business strategy information to other models through model transformations. Even though model transformations are out of the study's scope, quality can be assessed in this context from the characteristics of the models [56].

The following subsections specify how the above goals and context are translated into the experimental design. The package containing the training materials, experimental problems, instruments, and collected data can be accessed in [57].

#### Research questions and hypothesis formulation

To study model quality, we refer to the framework by Lindland et al. [58]. The framework identifies three quality concerns: 1. syntactic quality, which regards how well the model corresponds to the language, 2. semantic quality, which addresses how well the model corresponds to the domain, and 3. pragmatic quality, concerning how well the model corresponds to its audience interpretation. Considering our primary focus on analysing which language better represents the business strategy domain, the research questions for model quality will address semantic quality. According to Lindland, semantic quality regards how accurately a domain is represented in the model and how complete these models are. Accuracy and completeness have been included jointly in other experimental comparisons of model-driven methods [2–4]. Also, these quality goals are relevant for the secondary focus regarding organisational models as part of MDA-based development processes since transformable models are expected to contain all the correct and relevant statements about the domain [56].

To study the effects of the methods on the quality of the modelling process, we follow the method evaluation model of Moody et al. [59]. This model focuses on assessing the method users’ performance and their perception of its usefulness, ease of use, and intention to keep using it. Therefore, the research questions regarding the modelling process are referred to modellers’ efficiency and perceived satisfaction.

Based on the above frameworks, we define the research questions detailed below.

*RQ1: Is modelling accuracy affected by the modelling method used?* Following Lindland’s definition of semantic quality, we adhere to the definition of semantic accuracy in ISO25012^1^ as ‘the closeness of the data values to a set of values defined in a domain considered semantically correct’. The null hypothesis associated with this question is *H*_*a0*_—There are no differences in accuracy between LiteStrat and i* for business strategy modelling.

*RQ2: Is modelling completeness affected by the modelling method used?* According to Lindland, a model is complete if it ‘contains all the statements about the domain that are correct and relevant’ [58]. The null hypothesis to assert this question is: *H*_*c0*_—There are no differences in completeness between LiteStrat and i* for business strategy modelling.

*RQ3: Is modellers’ satisfaction affected by the modelling method used?* Following the model in [58], we consider the modeller’s satisfaction as the subjective perception of the method and the intention to use it for business strategy modelling. The null hypothesis formulated from these definitions is: Hs0—There are no differences in modellers’ satisfaction when using LiteStrat or i* for business strategy modelling.

*RQ4: Is modellers’ efficiency affected by the modelling method used? *Efficiency, another component of the model in [59], is defined as the effort required to apply a method. The null hypothesis to address this question is: *H*_*e0*_—There are no differences in modellers’ efficiency when using LiteStrat or i* for business strategy modelling.

The above research questions are consistent with the experimental comparison of model-driven methods presented in Sect. 2.1.

#### Factors and treatments

The factor under study is the modelling method, which has two treatments: i* and LiteStrat. Both treatments are applied to the representation of a business strategy case (the problem), which textually describes an organisation’s strategy in offering new products or services to its customers, harnessing opportunities, or mitigating environmental risks. The modelling methods will be applied without tool support. Subjects using LiteStrat must use the language’s constructs and relationships and follow the modelling procedure prescribed by the method. Subjects using i* must use the constructs and relationships of iStar 2.0 [55], but the modelling procedure depends entirely on the subjects’ criteria.

A second factor that might affect the observed phenomena is the problem to be modelled. The problem is considered a blocking variable since we are not interested in analysing differences between problems; however, we must ensure that the results are independent of the problems. This blocking variable has two levels: Problem 1 and Problem 2, which are further detailed in Sect. 2.3.4.

#### Response variables and metrics

To assess accuracy and completeness (RQ1 and RQ2), we will follow the approach of what we called *semantic inspections* in Sect. 2.1. The approach is to review the models designed by the subjects and inspect whether they accurately and completely represent the given problem. To do this, we divided the experimental problem’s sentences that present strategic information (namely *statements*) to construct the grading schemes for each problem.

For a more detailed analysis, we classified the statements into four types: (1) Motivation Statements, that describe the higher-level goals of the organisation and the elements from the organisation’s environment that drive such goals, (2) Action Statements, that describe what the organisation is willing to do to achieve its goals, (3) Roles and Responsibilities Statements, that quantitatively define the desired ends and their assignment to organisational roles, and (4) Outcome Statements, that describe the effects of the strategy among the organisation’s parts and on its environment.

To address RQ1, we define the *accuracy* response variable, meaning how well a statement in the problem description is represented in the conceptual model. Each statement in the experimental problem is checked and rated according to a three-level grading scale: 2 accuracy points if the whole statement is represented with the appropriate constructs and relationships provided by the language; 1 accuracy point if the statement is partially represented with the appropriate constructs, i.e., some constructs can be misused; and 0 accuracy points, if the statement is misrepresented or missing. It is worth noting that, for both languages, every combination of their concepts and relationships semantically valid is considered a correct representation. We define five accuracy metrics:

- *Motivation Accuracy (MA)*, the sum of accuracy points for all the motivation statements.
- *Actions Accuracy (AA)*, the sum of accuracy points for all the action statements.
- *Role-Responsibility Accuracy (RRA)*, the sum of accuracy points for all the role-responsibility statements.
- *Outcome Accuracy (OA)*, the sum of accuracy points for all the outcome statements.
- *Total Accuracy (TA),* the sum of all the above metrics: TA = MA + AA + RRA + OA

Taking into account the grading scale described above and the number of statements in each experimental problem (see Sect. 2.3.4), in Table 3 we detail the maximum scores for each variable and for each experimental problem. The grading scheme and the detailed evaluation of models are available in the experimental package [57]. In “Appendix”: Grading schemes, inspection guidelines, and solution examples for the experimental problems, we included the grading scheme and the semantic inspection guidelines for each problem, as well as a two reference models used during the assessment. We also included two models produced by the experimental subjects as examples.

**Table 3:** Maximum scores for accuracy metrics for each experimental problem

Accuracy metric	Max. accuracy score	
	Problem 1	Problem 2
MA	6	6
AA	6	6
RRA	8	8
OA	4	4
TA	24	24

For RQ2, we define the *completeness* response variable as the degree to which all concepts in a statement are represented in the model. For instance, the statement *‘the organisation x must achieve y*’ will be complete if the actor *organisation x* and the intention *y* are modelled and somehow related, using any of the constructs and relationships of the languages. It is worth noting that a statement can be complete, but, if the concepts and relationships used are not semantically valid, it will not be accurate. Similarly, we define a grading scale of two, one, and zero completeness points for complete, incomplete, and non-modelled statements, respectively, for the accuracy variable.

We also define five metrics associated with subsets of statements:

- *Motivation Completeness (MC)*, the sum of completeness points for all the motivation statements.
- *Actions Completeness (AC)*, the sum of completeness points for all the action statements.
- *Role-Responsibility Completeness (RRC)*, the sum of completeness points for all the role-responsibility statements.
- *Outcome Completeness (OC)*, the sum of completeness points for all the outcome statements.
- *Total Completeness (TC)*, the sum of all the above metrics: TC = MC + AC + RRC + OC

Since the completeness metrics have the same grading scale as accuracy metrics, the maximum values for MC, AC, RRC, OC, and TC are 6, 6, 8, 4, and 24, respectively, for both experimental problems.

For RQ3, we define the *user satisfaction* variable, which is addressed by the evaluation model proposed by Moody [59]. It defines three metrics: *Perceived Ease of Use (PEU)*, *Perceived Utility (PU)*, and *Intention to Use (IU)*. The model proposes a survey that consists of 16 questions expressed on a 5-point Likert scale, which represents the degree of satisfaction from totally disagree (1 point) to totally agree (5 points). The instrument provides six questions to measure PEU, whose scale is from six to 30 points (adding the results of the six PEU questions), eight questions to measure PU, whose scale is from eight to 40 points, and two questions for IU, whose scale is from two to 10 points.

Finally, to assess RQ4, we define the *efficiency* variable, measured as the *time* needed to perform the business modelling tasks. Time is measured in minutes and was self-reported by the subjects. We checked that the reported times were consistent from when the subjects receive the business strategy case to when they submit the finished models.

We summarise the variables and metrics for each research question in Table 4.

**Table 4:** Research questions, hypotheses, variables, and metrics

RQ	Hypotheses	Response variables	Metrics
RQ1	Ha0	Accuracy	TA, MA, AA, RRA, OA
RQ2	Hc0	Completeness	TC, MC, AC, RRC, OC
RQ3	Hs0	Satisfaction	PEU, PU, IU
RQ4	He0	Efficiency	Time

It is worth noting that motivation, action, role and responsibility, and outcome statements can be modelled with both i* and LiteStrat languages. Considering the example presented in Sect. 2.2 and the constructs detailed in the legend in Fig. 1, in Table 5 we provide examples on how the four type of statements can be modelled. Please note that for i*, a statement can be completely and accurately represented using other semantically valid constructs.

**Table 5:**
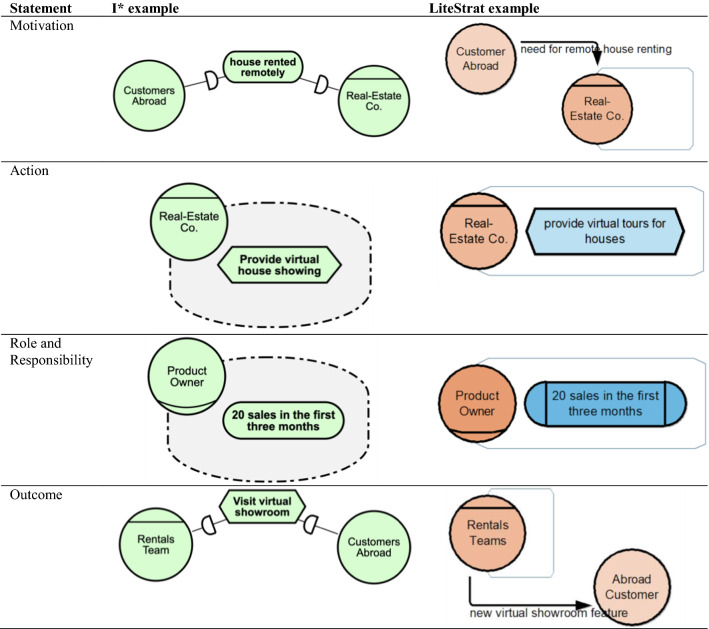
Examples of representations for motivation, action, role and responsibility, and outcome statements in i* and LiteStrat

#### Experimental problems

We designed two business strategy cases, describing a scenario where the organisations must define a strategy to address external factors. The cases detail the ends and means of the organisation and the organisational structure needed to deploy the strategy. Problem 1 describes a telecommunications company reacting to a new competitor with an improved service. Problem 2 describes an insurance company aiming to exploit a new business opportunity from a change in regulations. The experimental problems were paragraphs written in prose, in natural language, and the statements relevant for business strategy modelling were embedded in the narrative, so the subjects had to identify them from the text. The style and vocabulary used in the problems are based on business strategy cases from Forbes^2^ and McKinsey.^3^ To illustrate the style of the problem descriptions, an extract from problem 1 is given below. As can be seen, descriptions are general and are feasible to be modelled with both i* and LiteStrat.

… The company states that the Promotion Department should be involved in the design process of new insurance products from day one, and the Product Design Department should provide information from customer and competitor studies obtained during design. The Promotion Department must meet the objective of having the advertising campaign in place at least two weeks in advance of the product launch, which will be the responsibility of the Chief Publicist; in addition, its advertising campaigns must reach at least 20% of the market that is not yet a Short Life customer, which must be met by the Chief Publicist. The Insurance Design Department should ensure that customer and competitor research is carried out within a maximum of 20 working days, which is the responsibility of the Market Analyst….

The two problems have an equivalent complexity, with the same number of relevant statements for business strategy modelling. Each problem considered twelve statements, with 23 domain elements in Problem 1 and 24 in Problem 2, as detailed in the semantic inspection guidelines for Problem 1 and Problem 2 presented in Tables 13 and 14 in “Appendix”. It is worth noting that the problems’ text included statements that could be represented using the constructs of both LiteStrat and i*. The text does not include statements that could be only modelled in i*, such as qualities and resources. On the other hand, all the LiteStrat concepts can be represented using i* following the approach proposed in [24]. In “Appendix”, we provide LiteStrat and i* models of accurate and complete solution examples for Problem 1 and Problem 2. These elements and the complete problem descriptions, are available in the experimental package [57].

#### Experimental procedure

The experiment was performed in a remote, online context using ZOOM due to the COVID-19 pandemic. Initially, 30 subjects voluntarily participated in the activity. The procedure had two stages: the training session and the experimental activity. Each stage lasted 1 h.

The training session was performed just before the experimental activity, divided into two parts: a 20-min presentation introducing business strategy definitions and concepts and a second 40-min presentation addressing the modelling method. Both parts were taught by the instructor of the requirements engineering course, who is not involved in this article or in the design of LiteStrat. The first part was the same for all the subjects and covered general business strategy and organisation structure concepts. In the second part, the subjects were randomly assigned to LiteStrat or i* training sessions. The training for the i* group was performed in the first place, and then the second group received the LiteStrat training. For the i* group, besides presenting the framework, the training considered examples of how to use i* constructs to represent business strategy elements that are relevant to the experiment, based on the proposal presented in [24]. For the LiteStrat group, we presented the modelling language and procedure described in Sect. 2.2.

The experiment took one hour and consisted of three steps: completing the informed consent form, performing the modelling activity, and completing the post-test survey. The informed consent form and the post-test questionnaire were implemented in Google Forms, while the modelling activity was performed using pen and paper. During the modelling activity, the subjects had access to the experimental problems (randomly assigned and balanced between groups) and to the training materials. The subjects uploaded pictures of their models in the last question of the post-test questionnaire. All the subjects in the i* group completed the activity and the surveys, and two from the LiteStrat group dropped out. No models were discarded for lack of picture quality.

#### Experimental design

The experiment has a 2 × 2 factorial design [60], with four groups due to the combination of the two factors and problems. The subjects were randomly assigned to the groups. Discarding the subjects that dropped out, the final distribution of subjects is summarised in Table 6.

**Table 6:** Experimental design and distribution of subjects

	LiteStrat	i*
Problem 1	7	7
Problem 2	6	8

#### Experimental subjects

The 28 subjects were undergraduate students from a third-year Requirements Engineering course at the Universidad de Valparaíso. All of them had conceptual modelling background from a Software Engineering Foundations course which covered a subset of UML models (use cases, classes, state, sequence, and deployment diagrams). The course also introduced organisational goals and their relationship with requirements, although no modelling languages were introduced for this topic. None of the subjects had professional experience in software projects.

All subjects completed an informed consent form. Since participants were taking a course that included training in the two methods compared in the experiment, the study was considered non-interventional and therefore no ethics committee approval was required.

#### Data analysis

The models produced by the participants were reviewed using the grading scheme and inspection guidelines presented in “Appendix”. The first author graded the produced models, and the results were checked by the second author. The scores for each model and each statement are available in the experimental package [57].

We performed a univariate general linear model (GLM) analysis for each of the variables to analyse the interaction treatment*problem, thereby checking if there is a problem that works better for a specific treatment or, conversely, if the blocked design was successful. The assumptions needed to apply GLM were verified for all the variables using the Shapiro–Wilk test for the normality of the residuals and the Levene test for variance homogeneity. The analysis was performed using SPSS version 25. As Sect. 3 shows, one of the variables did not fulfil the normality assumption. Even though GLM is robust to normality deviation, we opted to apply the 1/√x transformation to data that are not normally distributed for the AC metric.

The results from the GLM are considered significant when the p-value is less than 0.05. The effect sizes are calculated for those metrics with significant results to analyse the magnitude of the differences using the partial η^2^ generated by SPSS; a value lower than 0.01 is considered to be a small effect, a value between 0.01 and 0.06 is associated with a moderate effect size, and higher than 0.14 is considered a large effect size. We also calculated the statistical power, which is the probability of rejecting the null hypothesis when it is false. Dyba et al. [61] report values greater than 0.39 for medium power and greater than 0.63 for large power.

## Results

In the following subsections, we present the results for the research questions stated in Sect. 2.3.1.

### Research Question 1: Accuracy

**Fig. 2:**
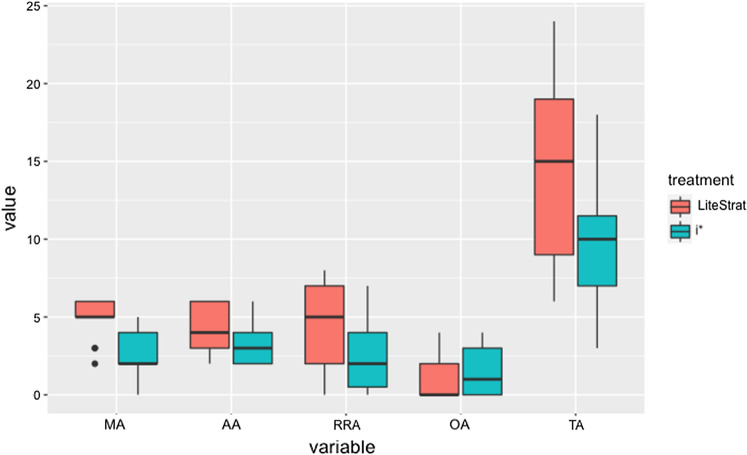
Box plot for the accuracy variables

To answer RQ1, we examined the Total Accuracy (TA) metric as well as the metrics for each requirement type: Motivation Accuracy (MA), Actions Accuracy (AA), Role and Responsibility Accuracy (RRA), and Outcome Accuracy (OA). Figure 2 shows the box plot for these metrics.

As can be observed, except for OA, LiteStrat shows better results for all of the accuracy metrics; the more pronounced differences can be identified in TA and in the MA.

The data analysis results for the accuracy metrics are detailed in Table 7. The Treatment column shows the probability value (p-value) for the effect of the treatment, which is our main interest and is presented in bold text. Statistically significant results for the treatment effect are marked with (**). The Interaction column shows the p-value of the Treatment*Problem interaction. The table also shows the means for LiteStrat and i*, the effect size (just for significant differences), and the statistical power.

**Table 7:** Data analysis for accuracy variables

Metric	Treatment	Interaction	Mean	Effect size	Power
TA	**0.014	0.409	LS:14.697 i*: 9.696	0.227	0.721
MA	**0.000	0.173	LS: 4.879 i*: 2.527	0.412	0.976
AA	0.117	0.788	LS: 4.036 i*: 3.179	−	0.346
RRA	0.051	0.125	LS: 4.488 i*: 2.473	−	0.506
OA	0.636	0.488	LS: 1.214 i*: 1.518	−	0.075

Significant differences were found for total accuracy (*p* = 0.014). The difference favours LiteStrat over i*, with a mean of 14.697 score points versus 9.696 points. The size of this effect can be described as large (es = 0.227), and the statistical power is high (*p* = 0.721). This result means that LiteStrat outperforms i* in the accuracy for describing the business strategy domain, and this difference has practical significance.

Concerning the analysis by requirement types, the MA variable also presents significant results (*p* < 0.001), favouring LiteStrat over i*, with a mean of 5.095 score points versus 2.661, respectively. The effect size is large (es = 0.412), and the statistical power is high (*p* = 0.976). This means that using LiteStrat is associated with better modelling of the external factors that trigger the strategy and a better specification of the overall goal that drives the organisational change. This difference has practical significance.

We did not find significant differences in the treatment effect for the rest of the metrics related to other requirement types, even though the statistical power is moderate for the AA and RRA metrics (*p* > 0.39). None of the metrics showed significant effects caused by the treatment*problem interaction.

From the above results, we can state that the null hypothesis H_a0_ can be rejected for the total accuracy (TA) and motivation accuracy (MA) metrics favouring LiteStrat over i*. For the Actions Accuracy (AA), Role and Responsibility Accuracy (RRA), and Outcome Accuracy (OA) metrics, the hypothesis H_a0_ cannot be rejected. We can also state that the results are independent of the problems since treatment*problem interactions are not significant.

### Research question 2: completeness

**Fig. 3:**
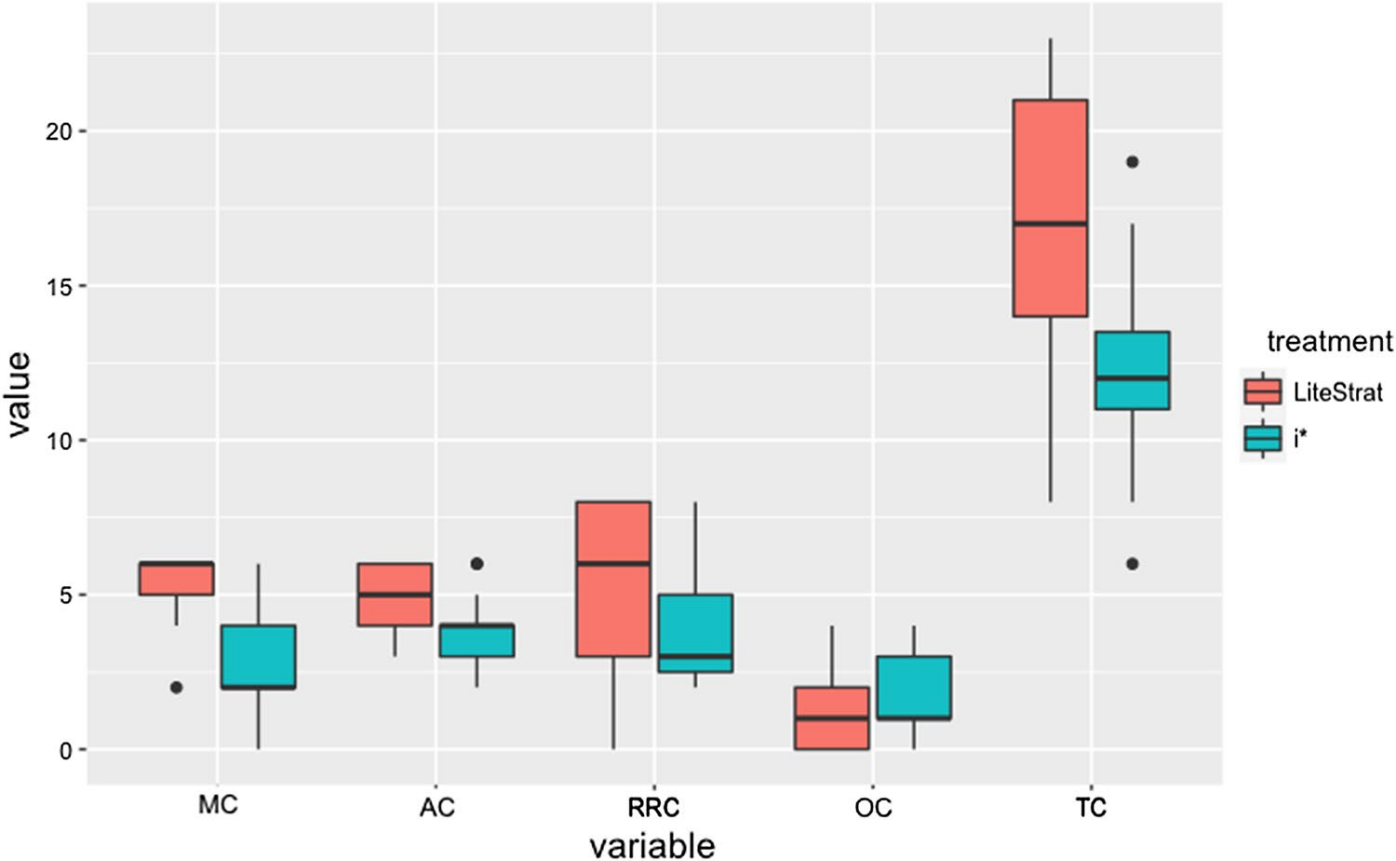
Box plot for the completeness variables

We conducted the same analysis presented in the previous section for the five metrics for completeness: Total Completeness (TC), Motivation Completeness (MC), Actions Completeness (AC), Role and Responsibility Completeness (RRC), and Outcome Completeness (OC). In Fig. 3, we present the box plot for the five metrics. As can be observed, most of the metrics favour LiteStrat over i*, except for OC. For TC, the results for i* seem to be more spread out than LiteStrat results, showing two mild outliers.

The data analysis results for the completeness metrics are detailed in Table 8. The treatment had significant effects for the TC, MC, and AC metrics. For TC, the p-value is 0.015, favouring LiteStrat over i* with a significant difference. The effect size is large (es = 0.222), while its statistical power is also high (pw = 0.710). For MC, the treatment is significant (*p* < 0.0001, favouring LiteStrat over i*. This effect size is large (es = 0.426), and the statistical power is high (pw = 0.981). For AC, the difference is significant (*p* = 0.046), also favouring LiteStrat over i*. The effect is large (es = 0.164), and the power is moderate (pw = 0.548). None of the metrics showed significant effects for the treatment*problem interaction (*p* > 0.05 in column Interaction).

**Table 8:** Data analysis results for completeness variables

Metric	Treatment	Interaction	Mean	Effect size	Power
TC	**0.015	0.208	LS: 16.381 i*: 12.268	0.222	0.710
MC	**0.000	0.153	LS: 5.095 i*: 2.660	0.426	0.981
AC	**0.046	0.296	LS: 4.821 i*: 3.884	0.164	0.548
RRC	0.178	0.414	LS: 5.179 i*: 3.929	−	0.265
OC	0.372	0.519	LS: 1.286 i*: 1.795	−	0.141

We did not find differences for the RRC and OC metrics, and the statistical power is low (*p*w < 0.39).

From these results, we conclude LiteStrat outperforms i* in terms of completeness of the business strategy models, and this difference has practical implications, given its large effect size. Following these results, the null hypothesis H_c0_ can be rejected for the Total Completeness (TC), Motivation Completeness (MC), and Actions Completeness (AC) metrics. For the Role and Responsibility Completeness (RRC) and Outcome Completeness (OC) metrics, H_c0_ cannot be rejected. We found no evidence that the experimental problems affected the results.

### Research question 3: efficiency

**Fig. 4:**
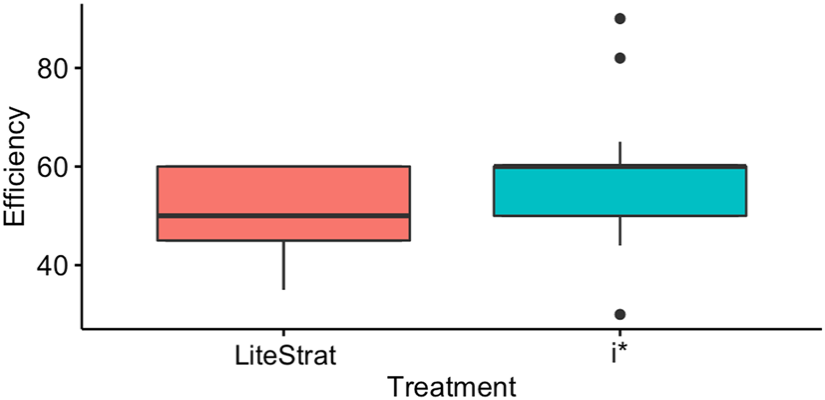
Box plot for efficiency

As shown in Fig. 4, the medians for LiteStrat show a higher efficiency for i*, as the top LiteStrat values are equal to the i* median. It is important to note that efficiency is measured in minutes, so lower values mean more efficiency. Note that all LiteStrat users completed the tasks in less time than the median of i* users.

The data analysis results for efficiency are shown in Table 9. The means show a tendency of the subjects to need less time to complete the modelling task using LiteStrat than using i* (50.214 min v/s 58.232 min, respectively). However, the difference is not statistically significant (*p* = 0.102). No significant interaction effects caused by the problem or by the treatment*problem interaction were found. The statistical power is low (pw < 0.39).

**Table 9:** Data analysis results for the efficiency variable

Metric	Treatment	Interaction	Means	Effect size	Power
Efficiency	0.102	0.336	LS: 50.214 i*: 58.232	−	0.372

Thus, it is not possible to reject the null hypothesis H_e0_, which means that differences were found in the effort needed to complete the modelling task using LiteStrat or i*. Also, the low statistical power does not allow us to suggest that there are no differences between the two treatments.

#### Research question 4: users’ satisfaction

**Fig. 5:**
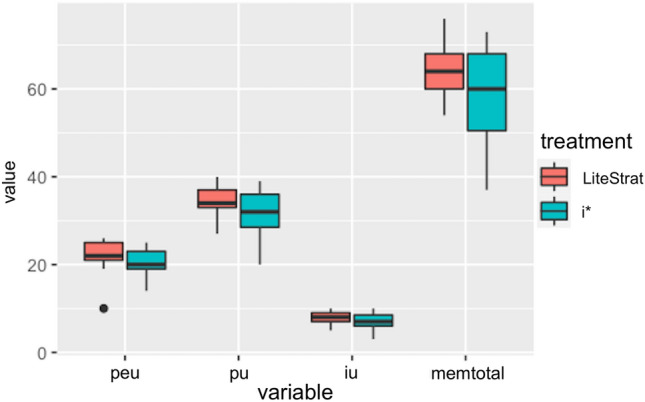
Box plot for users’ satisfaction

The user satisfaction survey followed the Method Evaluation Model, which defines three metrics: perceived ease of use (PEU), perceived usefulness (PU), and intention to use (IU). In Fig. 5, we present a box plot of the three metrics and the total survey score (MEM).

The data analysis results for efficiency are shown in Table 10. For the four metrics, no significant differences were found (*p* > 0.05), and the statistical power is low (*p*w < 0.39). No significant differences were found for the treatment and treatment*problem interaction.

**Table 10:** Data analysis for user’s satisfaction variables

Metric	Treatment	Interaction	Means	Effect size	Power
PEU	0.489	0.489	LS: 21.500 i*: 20.500	–	0.104
PU	0.387	0.154	LS: 34.548 i*: 31.705	–	0.293
IU	0.276	0.198	LS: 8.012 i*: 7.250	–	0.188
MEM	0.197	0.648	LS: 64.060 i*: 59.455	–	0.247

We did not find differences for perceived ease of use, perceived usefulness, and intention to use. So, the null hypothesis H_s0_ cannot be rejected.

## Discussion

This section describes the results’ implications and attempts to hypothesise the reasons for such results for each variable.

Since LiteStrat has been designed specifically for business strategy modelling while i* has a broader-scoped and guidance-free approach, LiteStrat users could produce more accurate and complete business strategy models, although i* could also be applied for this purpose [24]. Also, since LiteStrat introduces a structured modelling procedure, it could negatively affect modellers’ efficiency and satisfaction with the modelling process. Below, we analyse each of the metrics and discuss the deviation from the theorised results.

### Accuracy

The total Accuracy (TA) metric shows significant differences, favouring LiteStrat over i*. Since TA is the sum of the other accuracy scores, we discuss the results for the metrics composing TA below.

*The Motivation Accuracy Metric.* For the MA metric, a first factor that could explain the results is the *influence* relationship that exists in LiteStrat but not in i*, as shown in Table 2. Although i* users were taught to use goal or task dependencies to connect external actors with the organisation, the construct seems to be not considered by the subjects. Since influence does not imply an intentional action from the external actor to the organisation, it could be more appropriate than the i* relationship (*social dependency*). For example, a competitor that offers a new product does not *socially depends* on the organisation under analysis; it is more accurate to model that the competitor *influences* the organisation. The influence relationship might reduce the construct deficit [62] of i*, and therefore, improve the ontological completeness of LiteStrat over i*.

*The Role-Responsibility Accuracy Metric.* With a fairly significant difference (*p* = 0.051), the subjects more accurately modelled the assignment of responsibilities to roles with LiteStrat than with i*. Even though the difference between high-level and concrete ends was equally taught in training, LiteStrat users seem to have better support to model it. While in i*, subjects work with the goal construct, in LiteStrat, subjects work with goals and objectives, as detailed in Table 2. Having two different constructs could better support working with two levels of abstraction, which is consistent with the notion of construct deficit commented above. Another possible factor is using LiteStrat’s modelling guidelines, which guide users to connect objectives and roles [28]. Method engineering literature [63, 64] has widely supported the benefits of using guidelines.

**Fig. 6:**
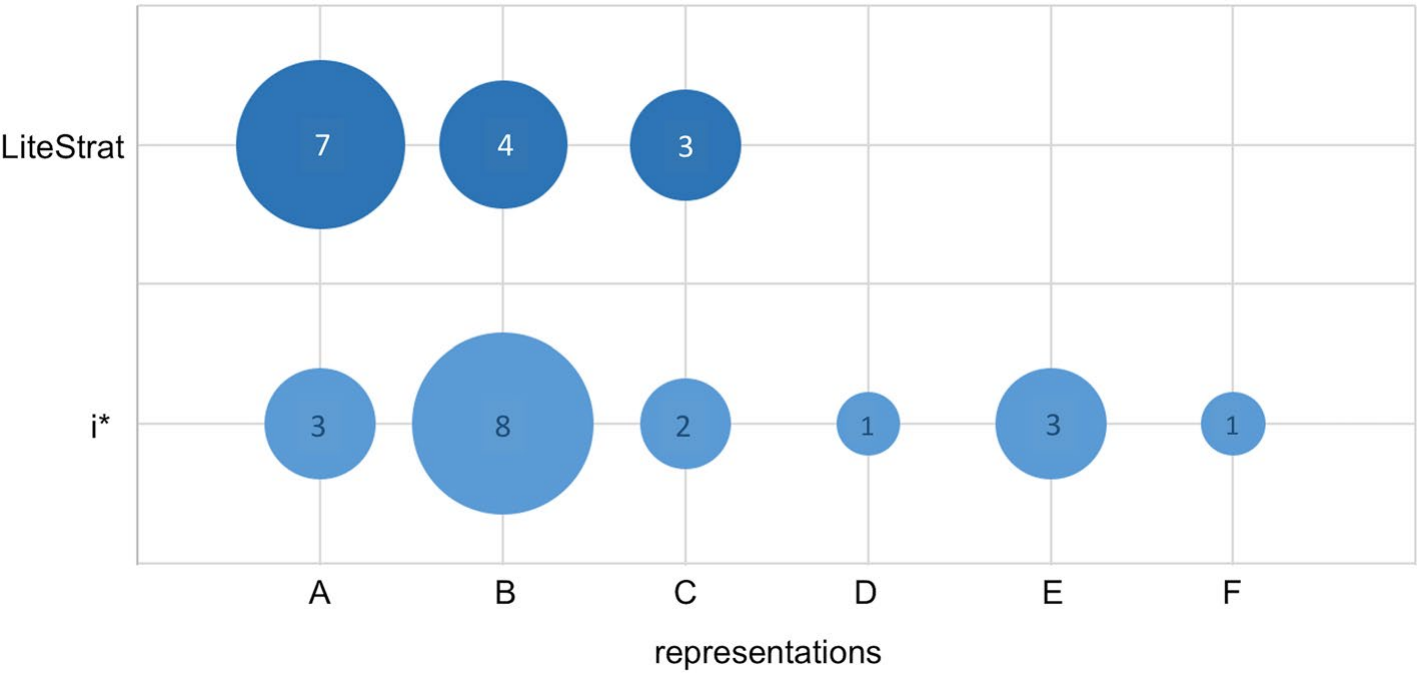
Number of subjects using different representations for role and responsibility assignment in LiteStrat and i*

Accurately modelling the assignment of responsibilities is of great interest in an MDD context since model transformation and alignment frameworks have exploited them as an integration point for goal and business process models [6, 54]. Typically, the assignment of responsibilities is modelled as dependencies between actors, which are transformed into (parts of) business process models that realise the collaboration of such actors to fulfil the dependency. We explored the models produced by the subjects looking for different representations of such statements. We found that i* users modelled these statements in six different ways, while LiteStrat users employed three different representations, as shown in Table 10. Figure 6 depicts the frequency of the different representations in LiteStrat and i*, using the A to F labels from Table 10. Although the more frequent representations in i* and LiteStrat could be helpful in an MDD context, when applying existing model-driven techniques, more information might be lost from i* models (even when they can be considered accurate). It is worth noting that in LiteStrat, only the first representation, “objective inside a role,” is considered accurate.

In conclusion, we believe that LiteStrat models could better support users to more accurately model strategic elements that can serve as integration points with other models at the CIM level (Table 11).

**Table 11:** Different representations for roles and responsibilities (R&R) in i* and LiteStrat. The subjects that not modelled roles or responsibilities are not included in the table

i*	Subjects											
R&R representation	19	27	28	29	1	3	6	8	11	16	25	26
A. goal refining a task	x				x						x	
B. goal dependency	x	x		x		x			x	x	x	x
C. quality qualifying a goal			x					x			x	
D. quality refining a goal							x					
E. quality qualifying a task										x	x	x
F. task											x	

LiteStrat	Subjects											
R&R Representation	2	4	5	7	9	10	12	14	15	18	23	24
A. objective inside role	x				x	x	x		x	x	x	
B. tactic inside role		x	x	x				x				
C. text inside role			x					x				x

*Actions accuracy metric* No significant differences were found for AA. As detailed in the experimental planning, the definition for Actions Accuracy (see Sect. 2.3.3) considered high-level and more specific strategic action statements. To get deeper insights on AA, we reviewed the results for each statement in Problem 1 that concerned the AA metric, and we classified them into high-level and detailed-level statements. An example of a high-level strategic action in the experimental Problem 2 is Leverage the strength of the app’s wide adoption to publish charges in detail and promptly, and a more specific action that follows the latter is to automate the charge validation process.

We did not find differences in accuracy for detailed-level statements. However, six out of seven subjects using LiteStrat got perfect scoring in high-level statements, while only three out of seven subjects got the perfect scoring using i*. We think that LiteStrat’s different constructs for high and detailed strategic actions (strategy and tactic, respectively) help improve accuracy since i* defines a unique construct for actions (Task), as shown in Table 2. The difference in action constructs can provide a semantic differentiation that guides users to identify these two strategic action levels in the text of the problems.

*Outcome accuracy metric* We conducted a similar analysis for the differences in the OA metric, which yielded non-significant differences. The analysis revealed that more subjects got the top OA score with i* (two subjects) than with LiteStrat (one subject). However, most of the subjects got poor scores with both methods. Since outcome modelling seeks to represent the outcomes of the strategy and its target actor or units, it seems that the LiteStrat construct for connecting actors (the influence) does not offer improvement over i*’s construct (the social dependency). We think that the name of the relationship can explain this (*influence*), which is not close to the outcome concept, so further improvements in the modelling guidelines or even a new relationship could be needed to represent better the strategy’s expected results.

### Completeness

Below, we discuss the metrics that compose Total Completeness (TC), starting with the ones that showed significant differences (Motivation Completeness and Actions Completeness) and then the non-significant ones (Role & Responsibility Completeness and Outcome Completeness).

*The motivation completeness metric* The MC metric yields the most significant differences among all the other metrics in the study, favouring LiteStrat over i* with a high statistical significance, high power, and large effect size.

We believe this is an expected result since the modelling procedure of LiteStrat explicitly addresses the identification of the external elements that trigger the business strategy and for modelling the overarching goal. Although i*’s constructs support modelling dependencies and strategic goals (as shown in training), its users seem unaware of the importance of these strategic elements. Together with significant differences in accuracy, this result supports the idea that LiteStrat improves the capture of knowledge of external forces that trigger the strategy.

*The actions completeness metric* Similarly to the Actions Accuracy metric, we believe that LiteStrat is better at helping the analysts to identify and represent strategic actions statements more completely than i* due to the differentiation between high-level and specific strategic actions. LiteStrat enforces this difference both in its modelling procedure and its modelling language.

It is worth noting that the completeness grading scheme for i* considers that different constructs can represent the organisation’s actions, even the *resource* and *quality* constructs. However, i* users may have overlooked differences between high-level and more specific actions since they did not have guidance (by an explicit modelling procedure) to recognise these differences.

To study the above idea further, we examined the resulting models for Problem 1 to explore if there are differences in the completeness between the high-level and the more specific strategic actions. The differences seem to be explained by the specific actions (25 points or LiteStrat and 20 for i*) rather than high-level strategic actions (11 points of completeness for LiteStrat versus 9 points for i*). We believe i* users could have overlooked the more specific actions since they considered them implicitly part of the more high-level action. Other authors have identified the lack of a concept for strategic actions in i*. For instance, Marosin et al. proposed to extend i* by adding the *key action* construct [65].

*The role and responsibility completeness metric* Unlike role and responsibility accuracy, no significant differences were found for RRC. It is worth noting that the grading scheme considers a statement as complete even if represented with inaccurate constructs. We believe that the i*’ users exploited its variety of constructs to represent somehow when a specific role must achieve an objective; however, these ad-hoc forms might hurt the understandability of the model and its transformation to other computing-independent models.

*The outcome completeness metric* There were no significant differences in Outcome Completeness between LiteStrat and i* users. We found that most of LiteStrat users missed to represent one of the two target actors in Problem 2 (current customers and new customers), as shown in Figs. 8, 9, 10 and 11 in “Appendix”. We believe that the subjects could have interpreted the training instructions that enforced modelling *the outcome of the strategy* in a literal way, i.e., that there is only one outcome (and target) of the whole strategy, thereby overlooking a second one.

### Users’ efficiency

Due to the lack of significant differences and the low statistical power, we cannot draw further conclusions about user efficiency. A possible explanation is the size of the sample (28 subjects); according to G*Power, the ideal sample size for achieving a statistical power high enough to find large effects is 29 subjects.

Another possible explanation is that the theoretical effect size may be low, making differences hard to see in practice.

On the one hand, LiteStrat is not specially designed to improve users’ efficiency, and the theoretical improvements may be low. On the other hand, i*’s free modelling approach may not be notably more efficient than LiteStrat’s guided modelling process, so differences may not be identifiable in a small sample size. This result guides us to think that LiteStrat could more accurately and completely represent business strategy without significantly affecting users’ efficiency.

### Users’ satisfaction

We did not find differences in user satisfaction metrics, and the statistical power was low. While the more rigorous and structured modelling procedure of LiteStrat may lead to think a worse satisfaction for LiteStrat, results yield that there are not significant differences.

The current results make us think that LiteStrat offers better accuracy and completeness without a critical, negative effect on users’ satisfaction. A more extensive study could help us identify whether significant and practical differences exist.

### Summary

We compared LiteStrat and i* to look for differences in the accuracy (H_a0_) and completeness (H_c0_) of the models, and users’ efficiency (H_e0_) and satisfaction (H_s0_). Considering that LiteStrat was specifically designed for representing business strategy while i* has a broader scope, the expected results were more accurate and complete models when using LiteStrat, while i* would perform better on users’ efficiency and satisfaction given its non-restrictive modelling procedure. Results confirm the differences favouring LiteStrat in models’ accuracy and completeness, whereas no differences for user’s satisfaction and efficiency were found. We found no evidence of the effect of the experimental problems in none of the results. However, problems with different complexity or less targeted in business strategy (e.g., mixing system requirements and business strategy concepts) could produce different results, which would be a matter of further study in industrial contexts.

**Fig. 7:**
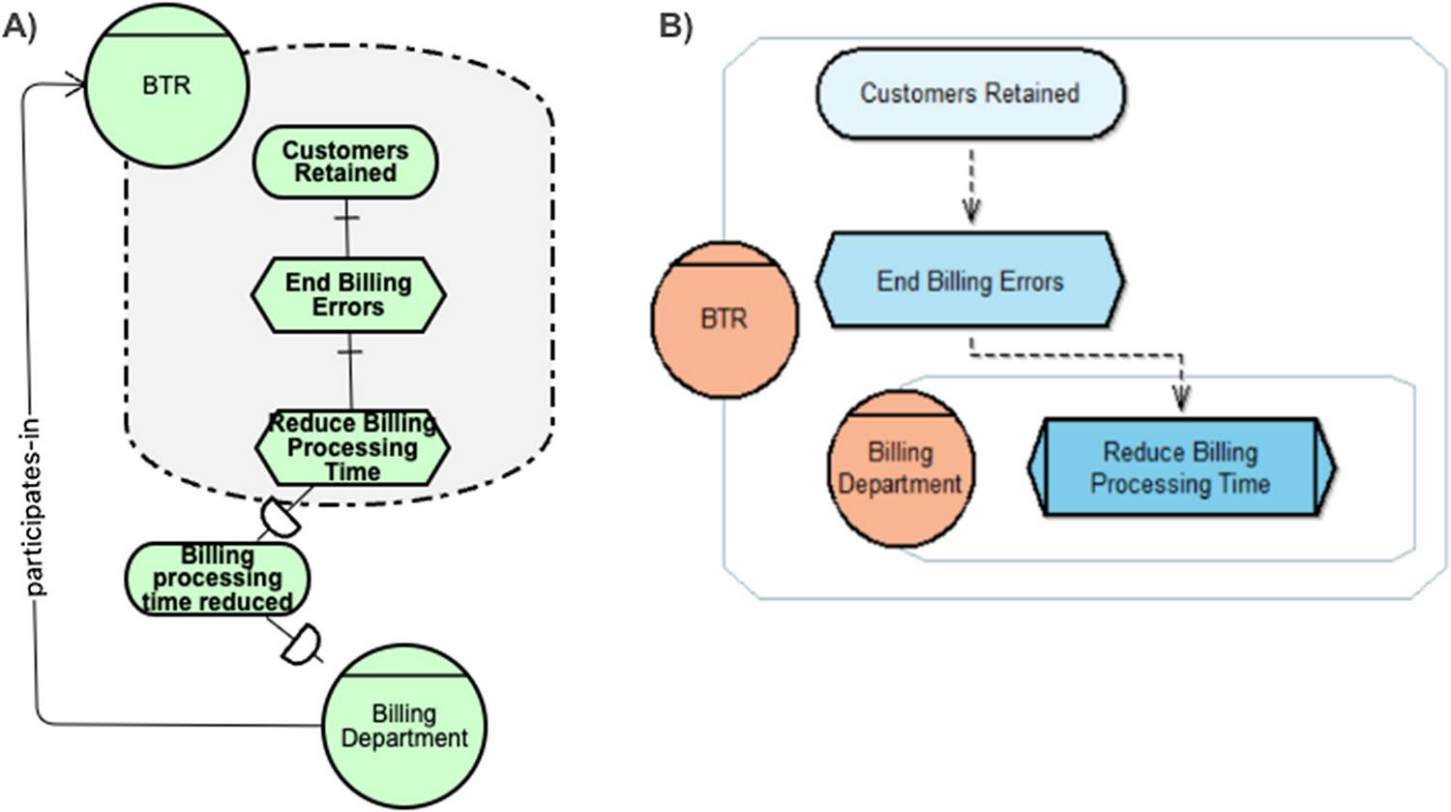
Number of subjects using different representations for role and responsibility assignment in LiteStrat and i*

**Fig. 8:**
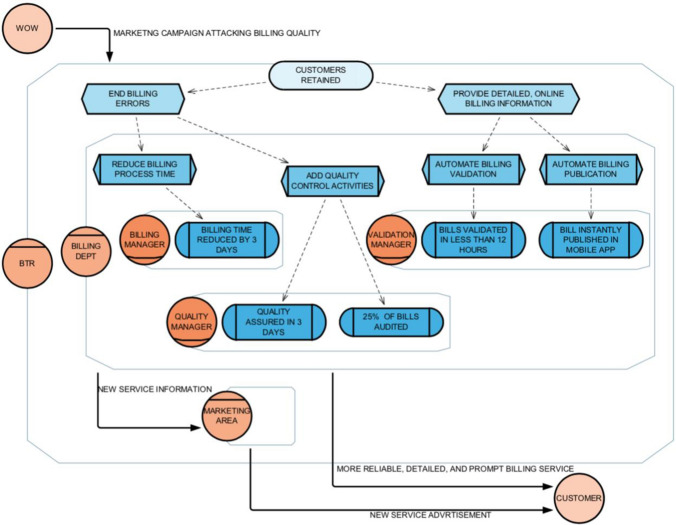
LiteStrat solution for Problem 1

**Fig. 9:**
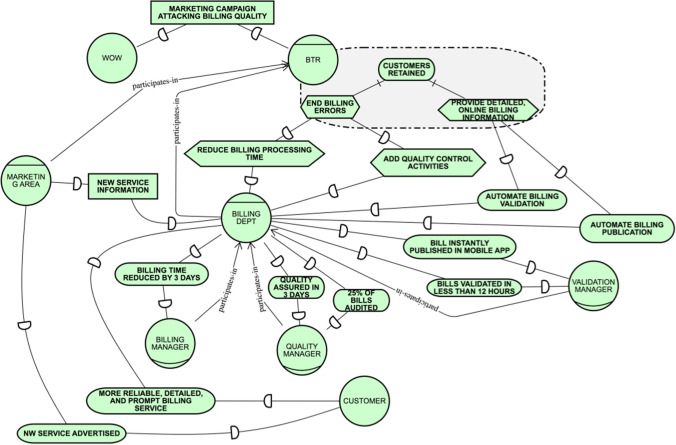
Example of a i* solution for Problem 1

**Fig. 10:**
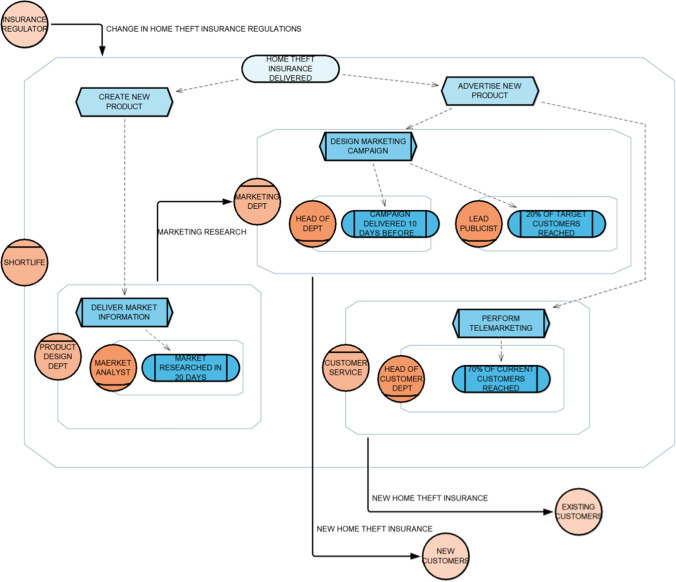
LiteStrat solution for Problem 2

**Fig. 11:**
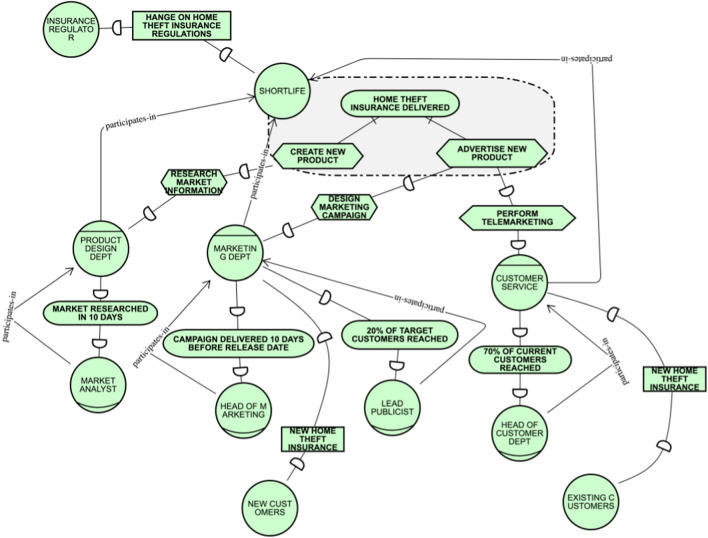
Example of a i* solution for Problem 2

Regarding the results on accuracy and completeness, the examples in Figs. 8 and 9 in “Appendix” could shed some light on the causes of LiteStrat our performing i*. As can be seen, the LiteStrat model seems to be simpler and more straightforward than the i* model, even though the two examples have the same number of domain elements, as detailed in Tables 13 and 14 in “Appendix”. We believe this is due to the languages' graphic representation of the refinements between goals and tasks and the participates-in links. Figure 7 illustrates these differences in a portion of a i* diagram (A) and a LiteStrat diagram (B). In LiteStrat, it is possible to have refinements of intentional elements across organisational units, such as "End Billing Errors" and "Reduce Billing Processing Time" in (B). At the same time, in i*, it is impossible to refine elements between actors for which a dependency link is needed, as in "Billing processing time reduced" (A). In LiteStrat, an organisation unit that participates in another is placed inside the parent organisation, and no link is needed, while in i* the participates-in link is needed. This fact may make LiteStrat models simpler and easier to design and manipulate for the domain under study.

After the above discussion, we believe that LiteStrat can be considered a valid alternative for (1) representing the information of the business strategy domain and (2) integrating business strategy information into an MDA context. The first claim is backed up by the significant differences in accuracy and completeness, where LiteStrat outperformed i*. LiteStrat’s specific constructs for business strategy and modelling procedure that empathises the identification of different levels of ends and means seem to serve modellers better to capture business strategy domain information without important efficiency and satisfaction trade-offs. The second claim is based on the fact that LiteStrat users followed a systematic approach, which produced a more homogeneous representation of the assignment of objectives to roles. These assignments are a point of integration with business process models in existing MDA frameworks [6, 66]. Since LiteStrat outperforms i* in modelling the cause of the strategic definitions (MA metric), LiteStrat provides the highest level of business information that drives the need for software development initiatives, contributing to fulfilling the purpose of the CIM level models in MDA-based methods.

### Threats to validity

This section presents the threats to the experiment’s validity and their associated mitigation actions. We classify the threats to validity according to Wohlin’s guidelines [60]. Below, we comment on the conclusion validity threats, which deal with the ability to draw the correct conclusions between the treatments and the outcomes of the experiment [60].

- Violated assumption of statistical tests: For each metric, we applied the Shapiro–Wilk test to check the normality of the residuals and the Levene test to determine variance homogeneity without finding any violations.
- Low statistical power: considering the sample size (28 subjects), there might be existing differences that we were not able to find. According to G*Power,^4^ a sample size of 39 subjects will be needed to find significant differences with a large effect size. Further replications of the study could help identify differences for the variables with non-significant results.
- Reliability of measures: We believe the measurement of the models’ accuracy has an unavoidable level of subjectivity. To mitigate this as much as possible, we performed a detailed review of related works to address this issue and decided to perform a semantic quality inspection like most experiments assessing modelling accuracy. The authors co-designed the grading scale, having in mind the modelling steps for both LiteStrat and i*. The solutions for the problems (see “Appendix”: Grading schemes, inspection guidelines, and solution examples for the experimental problems) were proposed by the first author and reviewed by the rest.
- Random heterogeneity of subjects: Working with undergraduate subjects with no industrial experience relevant to the experiment and with the same formation in conceptual modelling helped mitigate differences in experience that could impact the results.
- Random irrelevancies in the experimental setting: We designed the modelling task to be performed with paper and pencil to avoid technical differences in internet speed or power of the students’ computers. This also helped us to mitigate the threat of reliability of treatment implementation.
- Fishing: We mitigated the threat of searching for a specific result by defining a detailed protocol to rate the accuracy and completeness of the models. We also mitigated bias in the training process by asking a third party (the instructor of the requirements engineering course) to provide the training in LiteStrat and i*.

Internal validity threats deal with influences that can affect the independent variable without the researcher’s knowledge [60], which are commented on below.

- Instrumentation: We used standard metrics and instruments to measure user satisfaction and effort in method experimentation: the Method Evaluation Model survey [59] and time. We based our design on relevant and well-documented experiments to assess quality, presented in Sect. 2.1. The three authors participated and reviewed the grading scale and the solutions (see “Appendix” section).
- Selection: While working with undergraduate subjects might not represent the real population, it is considered a valid simplification in laboratory contexts for software engineering [67].
- Interactions with selection: This threat was mitigated by randomly assigning the subjects to the experimental groups and the treatments and problems to each group.
- Diffusion or imitation of treatments: We explicitly asked the subjects not to share the training materials or knowledge. We did not identify imitations of treatments during the data analysis.

Construct validity threats deal with an experimental design that does not reflect the theoretical constructs under study and are commented on below.

- Inadequate preoperational explication of constructs: We carefully studied and selected the metrics and variables other empirical studies used to assess the quality of the models, as presented in Sect. 2.1. We adapted it to the theoretical constructs as described in the experimental planning in Sect. 2.3.3.
- Mono-operation bias: This threat is related to confusing the treatment’s effect with the problems’ effect. We mitigated it by using two different experimental problems and verifying that the method and problem interaction did not affect the results. A possible threat regards the fact that the problems were specially designed for the experiment and are not taken from other third-party sources. We decided to opt for designing the problems to mitigate other relevant biases such as differences in complexity (that must be equivalent between the two problems), problem extension (we ensured that the problems can be modelled in the time slot given for executing the experiment), and that the relevant concepts for the MDD context (business strategy and organisational structure) were considered in the problem.
- Mono-method bias: This threat deals with having a unique measure for the effect of the treatments. We mitigated it by defining a set of metrics and a protocol to reduce the subjectivity in the scoring process.
- Restricted generalisability across constructs: This threat regards not studying other variables that can be negatively affected as a trade-off for the improvements of the new treatment. We mitigated it by assessing users’ satisfaction and efficiency, finding no drawbacks at a statistically significant level.

Social threats to construct validity regards the behaviour of the participants during the experiment, and their mitigation is commented below.

- Hypothesis guessing: This concerns subjects changing their behaviour after guessing the tested hypothesis or being afraid of being evaluated. This was mitigated by not providing information about the experiment’s goal or the relationship between the experimenters and the tested methods.
- Evaluation apprehension: We mitigated this threat by rewarding the subjects for their participation in the training and the experiment, regardless of their performance.
- Experimenter expectancies: Since the final aim is finding out which of the methods serves better for representing business strategy for its inclusion in an MDA-based method, all the metrics were collected as objectively as possible, giving no space to the results to be biased by the expectancies of the experimenter.

Finally, external validity threats, which concern the ability to generalise the study’s conclusions, have been managed in the ways commented below.

- Interaction of selection and treatment: This threat deals with the representativeness of the experimental subjects concerning the population. We have selected undergraduate computing science students, which, as the experimental software engineering community has widely discussed [67], is a valid simplification for testing and experimentally assessing novel techniques. However, we know that other empirical studies are needed to transfer the technique to specific industrial settings, such as technical action research [68].
- Interaction of setting and treatment: This threat deals with the representativeness of the setting and objects of study. We have mitigated it by designing problems that, even though they are not real-world problems, they are described similarly to strategic business cases in trending business magazines, such as Forbes.
- Interaction of history and treatment: The activity was performed on the same day and hour as the subjects usually have their lectures within the course content. No special events were identified previously or during the experimental activity.

## Conclusions and future work

Business knowledge modelling is of the uttermost importance for model-driven initiatives. Business strategy and organisational structure have become essential for evolving the organisations’ information systems design. While existing enterprise architecture modelling frameworks can represent these concerns, they have not been included in MDA-based methods for software development. Using existing goal modelling frameworks to represent business strategy could seem a valid alternative, especially if the frameworks have been included in MDA-based methods, such as i*. However, not having business strategy concepts could affect the accuracy and completeness of i* for representing business strategy. In the previous work, researchers have introduced LiteStrat, a modelling method based on i* to integrate organisational strategy information into an MDA-based development process.

This article presents an experimental comparison of i* and LiteStrat for representing business strategy. The experiment had a 2 × 2 factorial design, having the modelling method as the main factor of the study, with two treatments (i* and LiteStrat) and a secondary factor (the experimental problems) as a blocking variable. Twenty-eight undergraduate students performed a 60-min business modelling activity in an online, remote setting. The resulting models were semantically inspected to rate their accuracy and completeness. The modelling time was measured in minutes, and the user satisfaction was surveyed to determine subjects’ perception of each method according to ease of use, usefulness, and intention to use. The data were analysed using a univariate general linear model (GLM) analysis.

The experimental findings show that LiteStrat was more accurate than i* in representing business strategy. The main differences are explained by a more accurate representation of external factors that affect the organisation and the overarching goal that guides the organisational change. Also, LiteStrat models had better completeness than i* models, mostly due to a differentiated representation of the high-level organisational goals and strategic actions. No differences were found between LiteStrat and i* in terms of effectiveness and user satisfaction with the modelling method.

The significance of the experimental results was stated at the statistical level for both accuracy (*p* = 0.014) and completeness (*p* = 0.015). Internal and construct validity threats were carefully addressed during the experimental design and verified in the data analysis process. Conclusion and external validity threats were also mitigated. Note that the use of students is valid for evaluating novel methods even though these results may not be generalisable to experts in the industry.

The results confirm LiteStrat as a valid alternative for i* for business strategy modelling, with an improved model accuracy and completeness. The LiteStrat constructs, selected from business modelling frameworks, seem to effectively improve modelling business strategy modelling with respect to i*. LiteStrat’s influence construct could explain the more accurate representation of external events that trigger organisational goals and strategic definitions (e.g., competitor actions or customer needs), which could not be modelled as accurately with the social dependency constructs of i*. Furthermore, the LiteStrat modelling procedure seems to improve the completeness of the representation of strategic actions by guiding users to identify high-level actions and refinements to more specific actions (strategies and tactics, respectively). Finally, although no significant differences were found in the representation of more specific objectives and their assignment to roles, the variability in the representations of these elements suggests that LiteStrat guides its users toward a more homogeneous way of representing organisational roles and their responsibility. Having complete and accurate business strategy models is of great importance for connecting high-level business knowledge with more specific CIM level models, such as business process models, which makes LiteStrat a promising alternative for integrating business strategy information into an MDA-based development approach.

While further replications are needed to state that LiteStrat does not negatively affect users’ satisfaction and efficiency, the results guide us to think that subjects are not overwhelmed by LiteStrat’s modelling procedure, and there would be no trade-offs for improving models’ completeness and accuracy. Future work will focus on experimental replications with more experienced subjects and real-world business strategy case studies.

## Figures and Tables

**Fig. 12:**
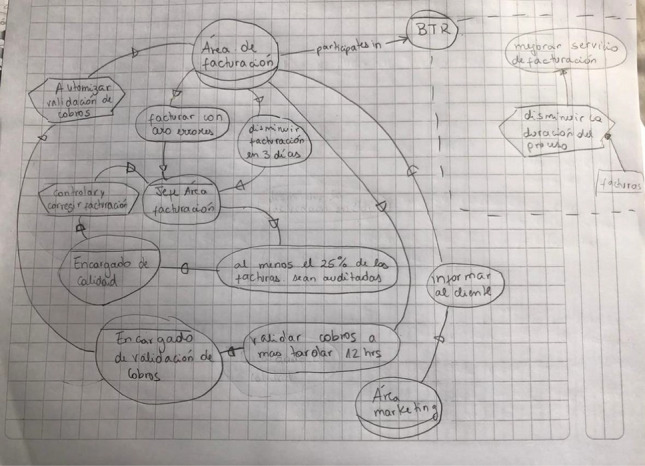
Example of an i* model designed by an experimental subject for Problem1

**Fig. 13:**
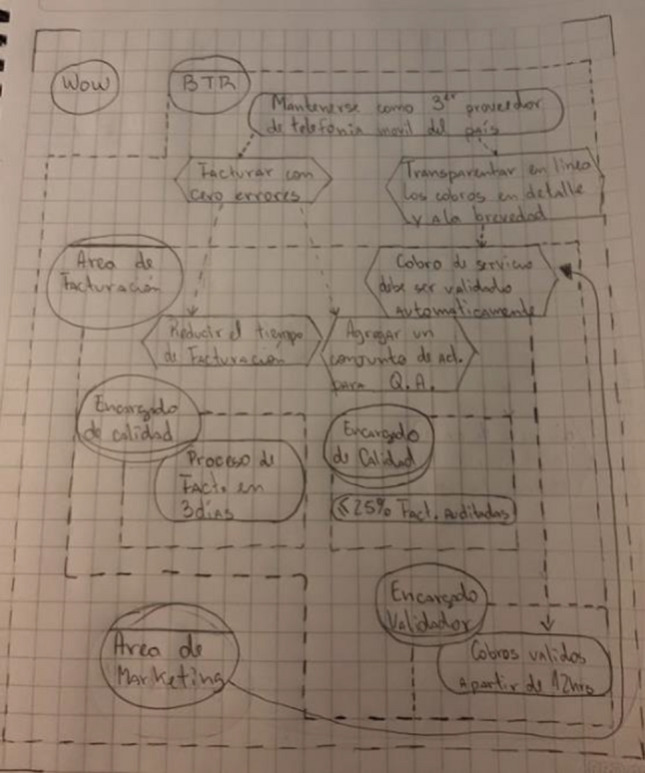
Example of a LiteStrat model designed by an experimental subject for Problem 1

## Data Availability

The data that support the findings of this study are openly available in ‘Zenodo’ at http://doi.org/10.5281/zenodo.7622995, in [57].

## Appendix: Grading schemes, inspection guidelines, and solution examples for the experimental problems

In Table 12, we present the accuracy and completeness grading schemes used to give marks for each statement in Problem and Problem 2. In Tables 13 and 14, we present the semantic inspection guidelines for assessing the statements. Figures 8, 9, 10 and 11 present i* and LiteStrat models as solution examples for Problem 1 and Problem 2, respectively. These solution examples would get full accuracy and completeness scores according to the semantic inspection guidelines, though alternative and equivalent solutions could also get full scores. We also provide an i* and a LiteStrat solution for Problem 1 provided by experimental subjects in Figs. 12 and 13, respectively.

**Table 12:** Accuracy and completeness grading schemes

*Accuracy grading scheme (per statement)*	
Achieved (2 point)	Subjects represent all the domain elements of the statement using the proposed or equivalent i*/LiteStrat Constructs
Partially Achieved (1 point)	Subjects represent most of the domain elements of the statement using the proposed or equivalent i*/LiteStrat Constructs
Unachieved (0 points)	Subjects represent most of the domain elements of the statement using constructs different to the proposed i*/LiteStrat constructs which are not equivalent
*Completeness grading scheme (per statement)*	
Achieved (2 point)	Subjects represent all the domain elements regardless of the i*/LiteStrat constructs used
Partially Achieved (1 point)	Subjects represent most of the domain elements of the statement, regardless the i*/LiteStrat constructs used
Unachieved (0 points)	Subjects fail to represent most of the domain elements of the statements

**Table 13:** Guidelines for the semantic inspection for Problem 1

Stmnt. type	Stmnt. Id	Domain elements	i* Concepts (example)	LiteStrat concepts
Motivation	P1R01	1. BTR	Agent or Actor	Organisation Unit
Motivation	P1R02	2. WOW	Agent or Actor	Actor
		3. "new marketing campaign"	Dependency (2 to > 1)	influence (1 to > 2)
Motivation	P1R03	4. Retain customers	Goal	Goal
Actions	P1R04	5. "End billing errors"	Task inside (1), refined from (4)	Strategy inside (1), refined from (4)
		6. "Detailed, transparent, and timely billing information"	Task inside (1), refined from (4)	Strategy inside (1), refined from (4)
		7. Billing Department	Agent or Actor that participates in (1)	Organisation Unit inside (1)
Actions	P1R05	8. "reduce the time needed to process invoices"	Dependency (5 to > 7)	Tactic refined from (5) inside (7)
		9. "add a set of quality control activities to the process."	Dependency (5 to > 7)	Tactic refined from (5) inside (7)
Actions	P1R06	10. Automatic Billing Validation	Dependency (6 to > 7)	Tactic refined from (6) inside (7)
		11. Automatic Billing Publication	Dependency (6 to > 7)	Tactic refined from (6) inside (7)
Roles and Responsibilities	P1R07	12. Billing Manager	Role that participates in (7)	Role inside (7)
		13. Reduce the billing time by 3 days	Dependency (7 to > 12)	Objective refined from (8) inside (12)
Roles and Responsibilities	P1R08	14. Quality Manager	Role that participates in (7)	Role inside (7)
		15. "Check and correct billing within 3 days"	Dependency (7 to > 14)	Objective refined from (9) inside (14)
Roles and Responsibilities	P1R09	16. "at least 25% of bills must be audited"	Dependency (7 to > 14)	Objective refined from (9) inside (14)
Roles and Responsibilities	P1R10	17. Validation Manager	Role that participates in (7)	Role inside (7)
		18. "the bills must be validated no later than 12 h after they have been made."	Dependency (7 to > 17)	Objective refined from (10) inside (17)
		19. "the publication of the bills in the app should be instantaneous"	Dependency (7 to > 17)	Objective refined from (11) inside (17)
Outcome	P1R11	20. Customers	Actor	Actor
		21. "re-engage your existing customers through better billing service"	Dependency (20 to > 7)	Influence (7 to > 20)
Outcome	P1R12	22. Marketing Area	Agent or Actor	Actor
		23. "inform customers about the new service"	Dependency (22 to > 20)	Influence (22 to > 20)

**Table 14:** Guidelines for the semantic inspection of Problem 2

Stmnt. type	Stmnt. Id	Domain elements	i* Concepts (example)	LiteStrat concepts
Motivation	P2R01	1. Short Life	Agent or Actor	Organisation Unit
Motivation	P2R02	2. Insurance Regulator	Agent or Actor	Actor
		3. "has determined that it is possible to add home theft insurance to car insurance. "	Dependency (2 to > 1)	Influence (1 to > 2)
Motivation	P2R03	4. "offer and advertise home burglary insurance, without recourse to third parties (marketing agencies)"	Goal inside (1)	Goal inside (1)
Strategic Action	P2R04	5. Marketing Department	Agent or Actor that participates in (1)	Organisation Unit inside (1)
		6. Product Design Department	Agent or Actor that participates in (1)	Organisation Unit inside (1)
		7. Design new product	Task refined from (4) inside (1)	Strategy refined from (4) inside (1)
		8. Advertise new product	Task refined from (4) inside (1)	Strategy refined from (4) inside (1)
Strategic Action	P2R05	9. Customer Service Department	Agent or Actor that participates in (1)	Organisation Unit inside (1)
		10. "Contact existing customers in to inform them of the new product"	Dependency (1 to > 9)	Tactic refined from (8) inside (9)
Strategic Action	P2R07	11. Design product marketing	Dependency (1 to > 5)	Tactic refined from (7) inside (6)
		12. provide information from customer and competitor studies	Dependency (1 to > 6)	Tactic refined from (7) inside (6)
Roles and Responsibilities	P2R06	13. Head of Customer Service Department	Role that participates in (9)	Role inside (9)
		14. "inform 70% of customers by telephone within the first week of a new product release."	Dependency (9 to > 13)	Objective refined from (10) inside (12)
Roles and Responsibilities	P2R08	15. Head of Marketing	Role that participates in (5)	Role inside (5)
		16. "have the advertising campaign in place at least two weeks in advance of product launch"	Dependency (5 to > 15)	Objective refined from (11) inside (15)
Roles and Responsibilities	P2R09	17. Lead Publicist	Role that participates in (5)	Role inside (5)
		18. the advertising campaigns should reach at least 20% of the market that is not yet a Short Life customer	Dependency (5 to > 17)	Objective refined from (11) inside (16)
Roles and Responsibilities	P2R10	19. Market Analyst	Role that participates in (6)	Role inside (6)
		20 "carry out the customer and competitor study within a maximum of 20 working days"	Dependency (6 to > 19)	Objective refined from (11) inside (19)
Outcome	P2R11	21.Customers	Agent or Actor	Actor
		22. offer the new theft insurance service	Dependency (21 to > 19)	Influence (19 to > 21)
Outcome	P2R12	23. New Customers	Agent or Actor	Agent or Actor
		24 to offer the new theft insurance service	Dependnecy (23 to > 19)	Influence (5 to > 23)

## Footnotes

1 https://iso25000.com/index.php/en/iso-25000-standards/iso-25012.

2 https://www.forbes.com/sites/mikalbelicove/2013/09/27/understanding-goals-strategies-objectives-and-tactics-in-the-age-of-social/.

3 https://www.mckinsey.com/capabilities/mckinsey-digital/our-insights/planning-in-an-agile-organization.

4 https://www.psychologie.hhu.de/arbeitsgruppen/allgemeine-psychologie-und-arbeitspsychologie/gpower.
